# 2025 AHA/ACC/AANP/AAPA/ABC/ACCP/ACPM/AGS/AMA/ASPC/NMA/PCNA/SGIM guideline for the prevention, detection, evaluation and management of high blood pressure in adults: a commentary from the European Renal Best Practice (ERBP)

**DOI:** 10.1093/ndt/gfag055

**Published:** 2026-03-10

**Authors:** Mehmet Kanbay, Sidar Copur, Pantelis Sarafidis, Charles J Ferro, Alberto Ortiz

**Affiliations:** Department of Internal Medicine, Division of Nephrology, Koc University School of Medicine, Istanbul, Turkey; Department of Internal Medicine, Koc University School of Medicine, Istanbul, Turkey; First Department of Nephrology, Hippokration Hospital, Aristotle University of Thessaloniki, Thessaloniki, Greece; Department of Renal Medicine, University Hospitals Birmingham, Birmingham, UK; Department of Cardiovascular Sciences, College of Medicine and Health, University of Birmingham, Birmingham, UK; Department of Nephrology and Hypertension, IIS-Fundacion Jimenez Diaz UAM, Madrid, Spain; Departament de Medicina, Facultad de Medicina, Universidad Autónoma de Madrid, Madrid, Spain; RICORS2040, Madrid, Spain

**Keywords:** cardiovascular events, chronic kidney disease, hypertension, mortality

## Abstract

American Scientific Societies (AHA/ACC and others) updated in 2025 the guideline on High Blood Pressure following 2024 ESC and 2023 ESH updates from Europe. Guidelines mostly agree on who should receive pharmacotherapy and on a common target of systolic blood pressure (SBP; <130 mmHg) and diastolic blood pressure (DBP) <80 mmHg for most, as well as on individualizing BP targets according to patient characteristics. However, they differ in nomenclature, pharmacotherapy for people at lower cardiovascular risk and aiming at SBP <120 mmHg. Overall, 2025 AHA/ACC supports pharmacotherapy earlier and achieving lower BP targets than other recent guidelines: it recommends [high cardiovascular disease (CVD) risk] or finds reasonable (not high CVD risk, if not corrected by lifestyle) initiating pharmacotherapy for hypertension, defined as SBP ≥130 mmHg or DBP ≥80 mmHg. The recommended (high CVD risk) or reasonable (not high CVD risk) target is SBP at least <130 mmHg, with encouragement to achieve SBP <120 mmHg, and DBP <80 mmHg. However, 2024 ESC and 2023 ESH use alternative terms (high normal or elevated blood pressure) for blood pressure values (i.e. ≥130–139 or ≥80–89 mmHg) that require pharmacotherapy only in high-risk groups. Additionally, 2023 ESH recommends avoiding actively aiming for SBP <120 mmHg or DBP <70 mmHg. 2024 KDIGO avoids the term hypertension and suggests a target SBP <120 mmHg for people with chronic kidney disease. It would be desirable that guidelines converge on such key issues as nomenclature and therapeutic targets.

## INTRODUCTION

Hypertension is the most common modifiable risk factor for multiple conditions including cardiovascular disease (CVD) such as coronary artery disease and heart failure, cerebrovascular events and dementia, as well as chronic kidney disease (CKD) [1, 2]. The 2025 Joint Guidelines of AHA/ACC/AANP/AAPA/ABC/ACCP/ACPM/AGS/AMA/ASPC/NMA/PCNA/SGIM (which we will refer to as the AHA/ACC guidelines) for the prevention, detection, evaluation and management of high blood pressure in adults were recently published [3], updating the 2017 document [4]. This update joins other recent updates, the 2024 European Society of Cardiology (ESC) guidelines for the management of elevated blood pressure and hypertension [5] and the 2023 European Society of Hypertension (ESH) guidelines for the management of arterial hypertension, endorsed by the International Society of Hypertension and the European Renal Association (ERA) [6]. We discuss the main points of the AHA/ACC guideline for diagnosis and treatment of hypertension in everyday clinical practice and compare these recommendations with recent European ones.

## GLOBAL DISEASE BURDEN OF HIGH BLOOD PRESSURE

Around 1.3 billion adults globally have arterial hypertension [7, 8]. The 2023 Global Burden of Disease (GBD) Study identified high systolic blood pressure (SBP) as the leading risk factor for global cardiovascular deaths, having a 3-fold higher impact than high low-density lipoprotein levels [1] (Fig. 1). High SBP was defined as >105–115 mmHg in adults older than 25 years of age. High SBP was also the global leading risk factor for attributable disability-adjusted life-years, following a nearly 22% increase from 2010 (Fig. 1) [9]. The negative impact of high SBP increases with aging (Fig. 1). Hypertension is also a modifiable risk factor for CKD progression and hypertensive kidney disease remains the second most common cause of incident kidney failure [10, 11]. Hypertension prevalence is expected to rise mainly due to aging of the global population as SBP tends to rise with increasing age due to multifactorial mechanisms including arterial stiffness and decreased kidney function, while diastolic blood pressure (DBP) tends to rise up until the fifth decade, plateaus for a decade and declines after that [12]. The World Health Organization estimated in 2023 that only 54% of hypertensive adults are diagnosed, with only 42% having received any treatment and only 20% of hypertensive adults reach adequate blood pressure (BP) control [13]. These numbers clearly highlight the need for improvements in prevention, awareness, diagnostics and treatment of hypertension.

**Figure 1:**
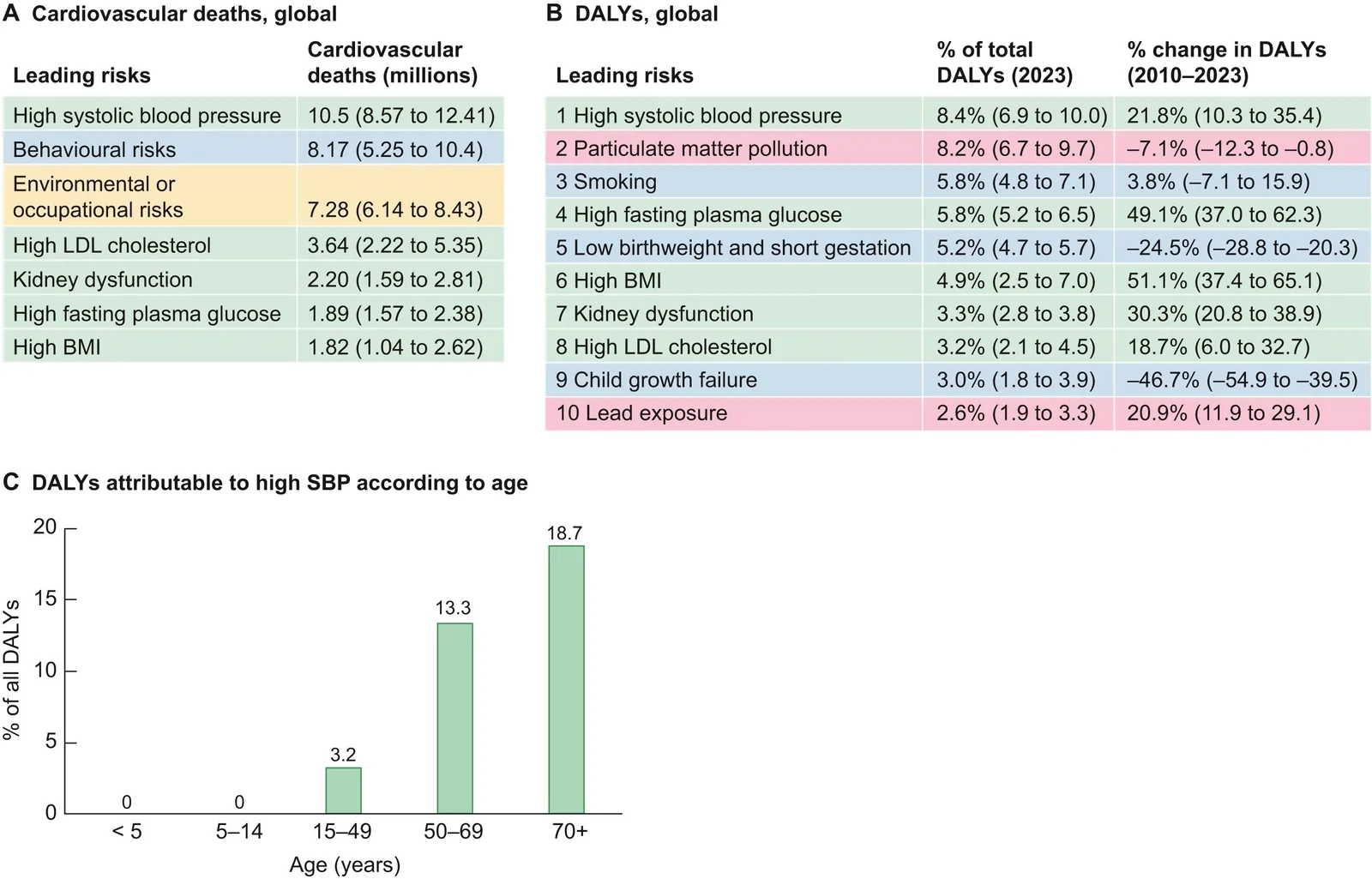
The leading causes of cardiovascular death and DALYs globally along with the changes in distribution between 2010 and 2023. Data from the 2023 Global Burden of Disease Study [1, 9]. Definitions: high SBP: >105–115 mmHg in adults >25 years of age; high LDL-cholesterol >0.9–1.4 mmol/L (35–54 mg/dL) in adults >25 years of age; high BMI: >20–22.5 kg/m^2^ in adults >20 years of age; high FPG >4.9–5.3 mmol/L (88–95 mg/dL) in adults >25 years of age; kidney dysfunction: eGFR ≤60 mL/min/1.73 m^2^. LDL, low-density lipoprotein; FPG, fasting plasma glucose; BMI, body mass index; LBW, low birth weight; SGA, short for gestational age; DALY, disability-adjusted life year.

## DEFINITION, STAGING, EVALUATION AND DIAGNOSIS

The 2025 AHA/ACC guidelines emphasize accurate and reliable BP measurement as the cornerstone of hypertension diagnosis and management. Office BP measurements, confirmed on at least two occasions, ambulatory BP measurement (ABPM) and home-based BP measurement (HBPM) are valid options recommended by the guidelines. Furthermore, the document emphasizes the superiority of ABPM and HBPM over office readings in terms of long-term clinical outcomes including CVD and all-cause mortality [14–16]. HBPM provides repeated measurements enabling dose titration for anti-hypertensive medications and offers statistically significant modest reductions in SBP and DBP without any other intervention [17, 18].

The three recent guidelines agree on the high risk conveyed by higher BP levels. However, they differ in guideline name, nomenclature for BP categories (optimal, normal, high-normal, non-elevated and elevated BP; the same term is used for different cut-off points) and definition of hypertension [3, 5, 6]. This heterogeneity and the lack of a common language do not facilitate communication with society, patients and medical students, or between physicians. While it is helpful that both European guidelines share the same concept of hypertension (SBP ≥140 or DBP ≥90 mmHg), the lack of global consensus (2025 AHA/ACC define hypertension as SBP ≥130 or DBP ≥80 mmHg, as did the prior 2017 guidelines) makes it difficult to transmit which populations are enrolled in clinical trials and to whom the results apply. Once more, the need to share a common language should be emphasized. International guidelines, such as the 2024 KDIGO guideline on CKD, have opted to avoid the term hypertension when discussing treatment decisions [19].

All hypertension guidelines evaluated potential advantages and disadvantages of BP measurement methods. While the 2025 AHA/ACC guidelines did not comment on BP staging based on ABPM or HBPM (also termed self-measured BP monitoring), they provided corresponding BP values between methodologies (Fig. 2) and strongly recommended out-of-office BP measurements for hypertension diagnosis or exclusion of white-coat or masked hypertension unless the patient has in-office SBP ≥160 mmHg or DBP ≥100 mmHg. Similarly (Class I recommendation, Level A of evidence), it emphasizes the need for out-of-office BP measurement to evaluate the response to anti-hypertensive therapy. These recommendations are based on studies demonstrating lack of increased CVD risk among patients with white-coat hypertension compared with normotensive individuals and studies indicating similarly high risk for patients with masked hypertension and patients with sustained hypertension [20, 21]. Patients should be screened for masked or white-coat hypertension as their diagnosis is highly likely to alter management and prevent unnecessary treatment and treatment-related adverse effects including orthostatic hypotension and hypoperfusion-related adverse effects among patients with white-coat effect. Diagnosis of masked hypertension may improve the therapeutic approach and prevent hypertension-mediated end-organ damage (HMOD). ABPM is endorsed by all recent guidelines as an effective, valid and cost-effective method for hypertension diagnosis and adjustments of anti-hypertensive medications [22]. HBPM enables more frequent measurements with improved treatment decisions and empowerment of patients in the decision-making process, leading to higher patient adherence [23].

**Figure 2:**
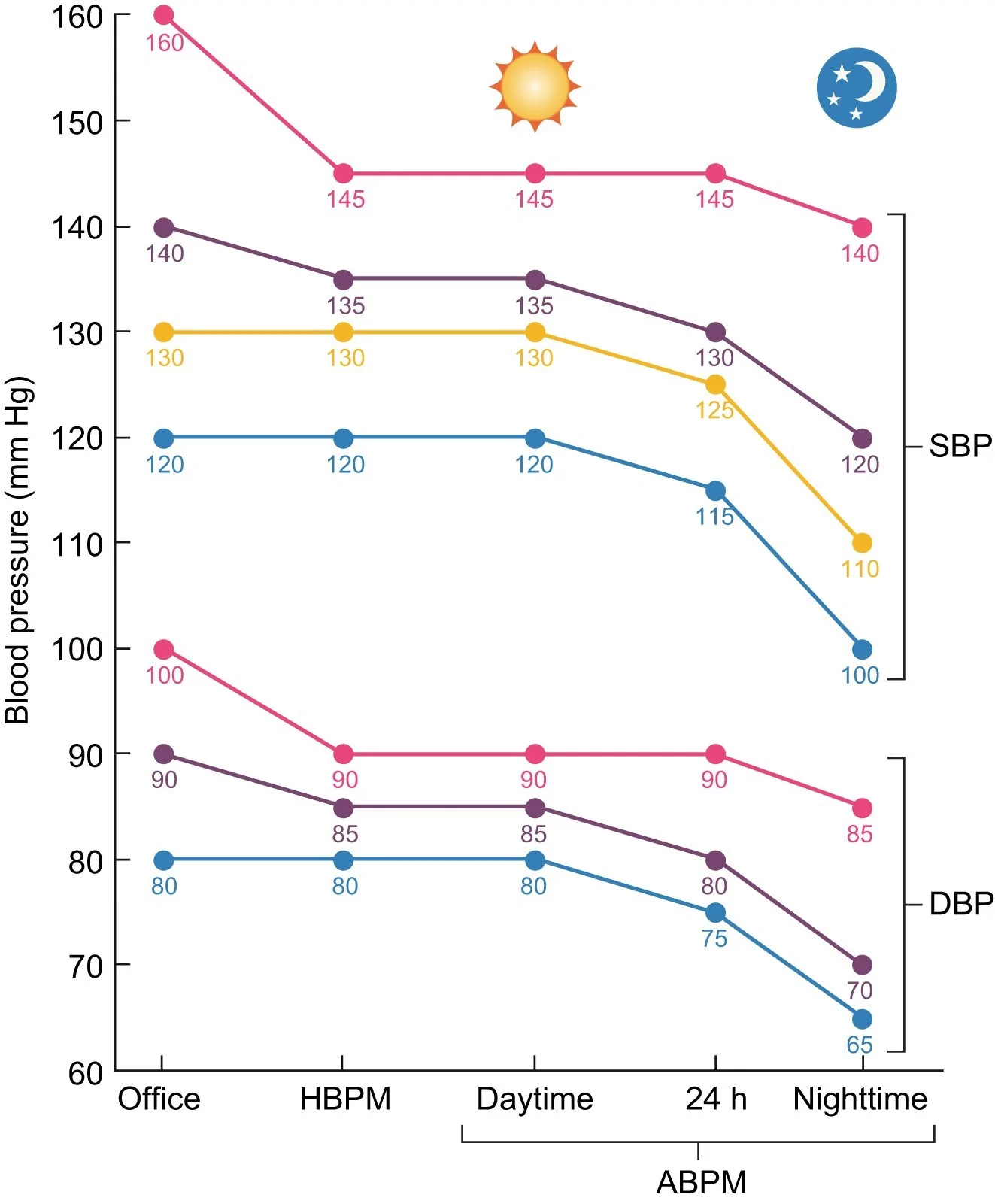
Values of SBP/DBP for HBPM and ABPM corresponding to office SBP and DBP levels. Note that HBPM and daytime ABPM share the same values. Graph designed using data from Table 7 in Jones *et al*. [3].

The 2025 AHA/ACC guidelines do not specifically discuss patient groups in which diagnosis and treatment of high BP may be challenging, including atrial fibrillation, in contrast to the 2023 ESH and the 2024 ESC guidelines. Consideration of such factors while evaluating BP readings is important, as atrial fibrillation may lead to falsely elevated or decreased BP readings with oscillometric measurements.

The 2025 AHA/ACC guidelines recommend measurement of urine albumin-to-creatinine ratio and protein-to-creatinine ratio in all newly diagnosed hypertension patients as routine laboratory workup, with Class I recommendation and Level C of evidence based upon expert opinion consensus, which was classified as an optional laboratory diagnostic tool in the previous 2017 AHA/ACC guideline. Presence of even microalbuminuria among hypertensive patients has been associated with poor clinical outcomes including higher rates of major adverse cardiovascular events including myocardial infarction and stroke, kidney disease progression and all-cause mortality [24]. Such revised recommendation is clinically significant and may shed light upon therapeutic choices, as micro- or macro-albuminuria may indicate HMOD leading to preferential use of renin–angiotensin–aldosterone system (RAAS) inhibitors.

## SECONDARY HYPERTENSION

Evaluation should assess HMOD and secondary hypertension. Secondary hypertension is now considered more common than previously assumed, affecting 10%–35% of all hypertensive adults. The 2025 AHA/ACC guideline mentions its higher prevalence among patients with stage II hypertension (SBP ≥140 or DBP ≥90 mmHg), treatment-resistant hypertension, loss of BP control in a previously regulated hypertensive patient, early-onset hypertension before the age of 30 years, or HMOD disproportionate to the duration or severity of hypertension. The 2025 AHA/ACC guideline emphasizes the low rates of screening for secondary hypertension despite the high prevalence of such phenotypes along with potentially marked improvements in BP and CVD risk with targeted therapies. The most common causes for secondary hypertension include CKD, primary hyperaldosteronism, renovascular hypertension and obstructive sleep apnea [25]. Routine laboratory testing for new diagnosis of hypertension should include, among others, estimated glomerular filtration rate (eGFR) and the urinary albumin-to-creatinine ratio to assess for CKD. Like the ESC guidelines, the 2025 AHA/ACC guideline recommends screening for primary hyperaldosteronism, without necessarily stopping antihypertensive medications except for mineralocorticoid receptor antagonists (MRAs), in patients with suspected secondary hypertension and/or resistant hypertension even when hypokalemia is absent (Class 1 recommendation, Level B of evidence), to improve diagnostic and therapeutic approaches. Even though higher screening rates for secondary causes may potentially increase the number of false-positive results requiring confirmatory tests (for primary aldosteronism, these include aldosterone suppression tests and invasive procedures such as adrenal venous sampling coupled with higher medical expenses), a correct diagnosis may significantly alter the prognosis and treatment. The diagnosis of primary hyperaldosteronism may lead to unilateral adrenalectomy in suitable patients with unilateral aldosterone over-secretion or MRA therapy in bilateral disease.

## PREVENTION OF HYPERTENSION

The 2025 AHA/ACC guideline contains the term “prevention” in the title and a brief section on prevention of hypertension which states that lifestyle changes recommended to treat hypertension will also prevent hypertension. This sends a short but powerful message: we should not wait until disease develops to implement a healthy lifestyle. The 2024 ESC also contains a section on prevention which is lacking from 2023 ESH guidelines, although the latter do indicate that a heart-healthy lifestyle is a fundamentally important approach to prevent or delay the onset of hypertension. Coincidentally, 2025 AHA/ACC and 2024 ESC use the term “elevated” to refer to SBP 120–129 mmHg, while the 2023 ESH guideline calls it normal. Overall, Nephrology should learn from Cardiology to emphasize prevention and early action, even if limited to a healthy lifestyle. While the 2023 ESC guidelines on the treatment of heart failure have a specific chapter on prevention that recommends sodium-glucose cotransporter 2 (SGLT2) inhibitors and finerenone to prevent heart failure in people with type 2 diabetes and CKD [26], the 2024 KDIGO guidelines lack recommendations on prevention of CKD [19]. However, an interest in prevention, including the definition of pre-CKD stage akin to elevated or high-normal hypertension, is starting to develop momentum as the starting point to proactively prevent CKD [27, 28].

## THERAPEUTIC APPROACHES: LIFESTYLE AND PHARMACOTHERAPY

All the guidelines explicitly and strongly emphasize lifestyle modifications for the entire population, irrespective of their BP status. Reduction of dietary sodium intake below 2300 mg/day (Class I recommendation, Level A of evidence), or ideally below 1500 mg/day, use of potassium-based salt substitutes unless CKD or another potentially hyperkalemia-inducing condition is present (Class 2A recommendation, Level A of evidence), healthy diet [such as the Dietary Approaches to Stop Hypertension (DASH) diet], alcohol abstinence (Class I recommendation, Level A of evidence), physical activity (Class I recommendation, Level A of evidence) and weight reduction of at least 5% in overweight or obese individuals (Class I recommendation, Level A of evidence) are recommended even if hypertension is absent. Lifestyle modifications lead to considerable reductions in BP levels and reduce CVD risk [29–31].

The use of pharmacotherapy for the management of hypertension according to BP levels, presence of comorbidities and cardiovascular risk has been addressed by the three recent guidelines, and although there was broad agreement in some core issues, they reached different conclusions on other also important issues, such as the BP threshold to initiate pharmacotherapy in lower cardiovascular risk patients as well as on the aim of lowering SBP below 120 mmHg (Fig. 3).

**Figure 3:**
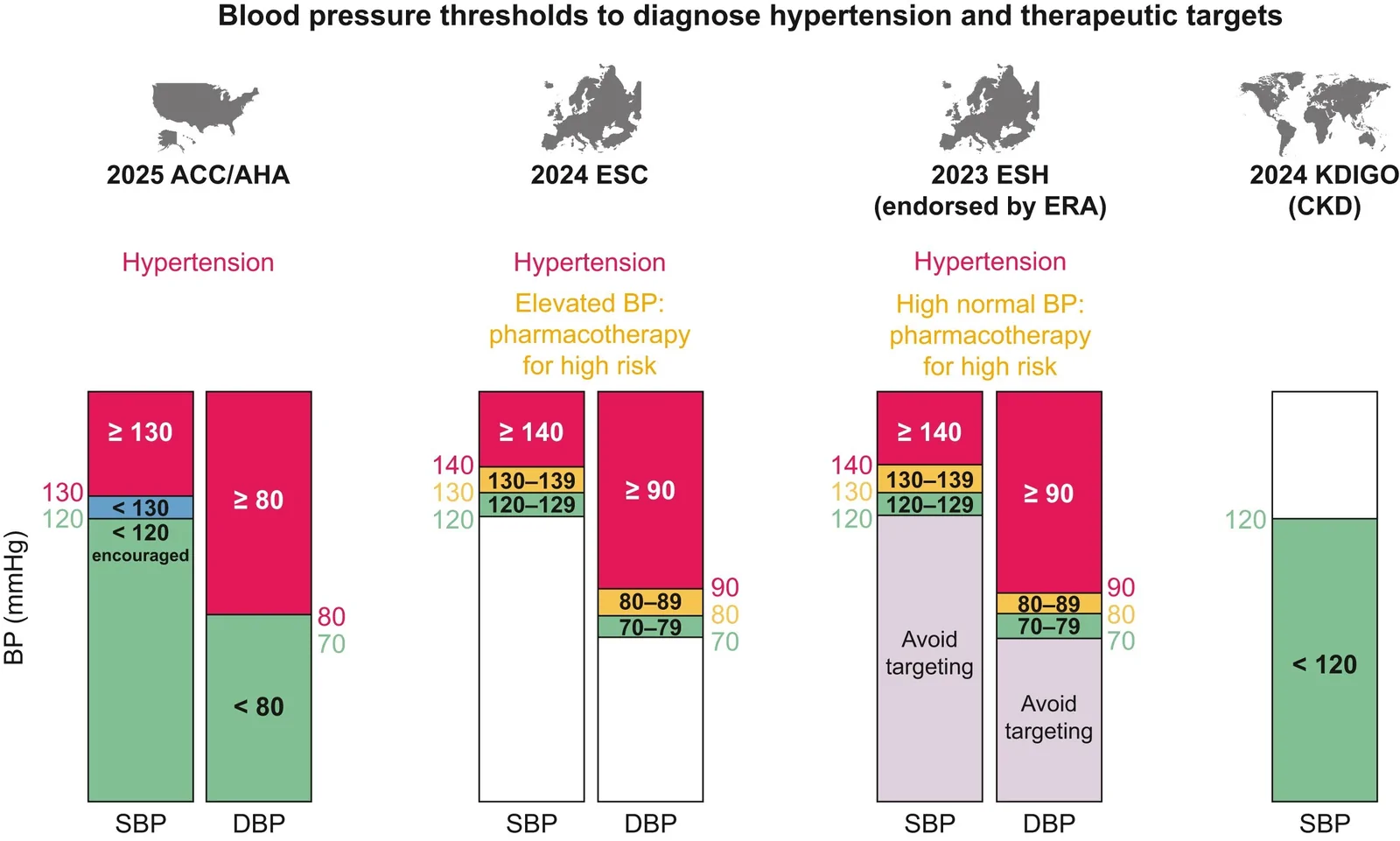
BP thresholds to diagnose hypertension and therapeutic targets in recent clinical guidelines for CKD patients. All guidelines basically agree on who should be treated with medication for high BP and agree on a common target for SBP (<130 mmHg) and DBP (<80 mmHg) for most, and that therapy and BP targets should be individualized according to clinical situation, cardiovascular risk, and patient frailty or age. However, there are differences in nomenclature and on the recommendation for aiming to lower SBP below 120 mmHg. The 2025 ACC/AHA guideline considers hypertension at any SBP/DBP level that may require treatment with pharmacotherapy (i.e. ≥130 or ≥80 mmHg) and recommends pharmacotherapy for hypertension independently of the presence of high cardiovascular risk. However, 2024 ESC and 2023 ESH use alternative terms (high normal or elevated BP) for BP values (i.e. ≥130–139 or ≥80–89 mmHg) that require pharmacotherapy in high-risk groups. 2024 KDIGO avoids any controversy by avoiding the term hypertension altogether, emphasizing that BP targets refer to standardized office BP measurement. Color coding of images: burgundy represents hypertension, orange represents elevated or high normal BP where pharmacotherapy is recommended for people at high cardiovascular risk; blue represents minimal recommended BP target for pharmacotherapy; green represents desirable or encouraged BP target for pharmacotherapy; pink represents a BP target that should be avoided to actively pursue with pharmacotherapy.

In the ESH 2023 guidelines, timing of initiation of pharmacotherapy treatment depends on office BP levels, presence of symptoms, presence of HMOD, CVD or high risk of CVD, and cardiovascular risk estimation with the Systematic Coronary Risk Evaluation 2 (SCORE2) algorithm for other patients. In asymptomatic patients, without HMOD, CVD or CKD, initiation of lifestyle measures is recommended for patients with office BP between 140–150/90–95 mmHg, followed by pharmaceutical therapy if BP is not controlled at 3 months, while immediate start of antihypertensive treatment is recommended for those with BP ≥150/95 mmHg or with a diagnosis of hypertension (office BP levels ≥140/90 mmHg) and symptoms, HMOD or CVD/CKD [6]. However, they also recommend drug treatment initiation with high normal BP (defined as office SBP ≥130 or DBP ≥80 mmHg) in patients with CVD. The general goal is to lower office BP to <130/80 mmHg, especially in younger (<65 years) people and, if tolerated, in older people, although the target for older people may be higher. In general, they recommended not to actively aim for office SBP <120 or DBP <70 mmHg.

The 2024 ESC guidelines recommend anti-hypertensive treatment initiation after diagnosing hypertension (SBP ≥140 mmHg and/or DBP ≥90 mmHg) or in patients at high risk with elevated BP (defined as SBP ≥130 mmHg and/or DBP ≥80 mmHg). High-risk patients include those with CKD (eGFR <60 mL/min/1.73 m^2^ or urine albumin-to-creatinine ratio ≥30 mg/g), established CVD, HMOD, diabetes mellitus or familial hypercholesterolemia, with 10-year fatal and non-fatal CVD risk score over 10% assessed via SCORE2 for age 40–69 years and SCORE2-Older Persons for older patients [32, 33]. Patients with 10-year risk score of 5%–10% may be further examined via additional risk factors including family history, ethnicity, HIV, autoimmune disorders, pregnancy complications including pre-eclampsia or gestational diabetes, or evaluation of coronary artery calcium score or pulse wave velocity or Doppler ultrasound for carotid or femoral atherosclerotic plaques. When pharmacological intervention is initiated, the BP target is 120–129/70–79 mmHg, being cautious in adults with orthostatic hypotension, moderate-to-severe frailty, limited life expectancy and age ≥85 years.

The 2025 AHA/ACC guidelines use an estimated 10-year CVD risk ≥7.5% as cut-off for high-risk definition based on the American Heart Association Predicting Risk of CVD EVENTs (PREVENT) equations developed in the USA [34, 35]. The 7.5% threshold is equivalent of 15% cut-off for Framingham Risk Score [36, 37]. Additional high-risk patients include those with established CVD, CKD and diabetes mellitus. Initiation of pharmacological therapy is recommended (high CVD risk) or found reasonable (not high CVD risk, if not corrected by lifestyle) for all people with hypertension, defined as SBP ≥130 mmHg or DBP ≥80 mmHg. The recommended (high CVD risk) or reasonable (not high CVD risk) target for all groups is SBP at least <130 mmHg, with encouragement to achieve SBP <120 mmHg and DBP <80 mmHg. Overall, 2025 AHA/ACC recommends starting pharmacologic treatment earlier and having lower BP targets than other recent guidelines. It acknowledged that shared decision-making by clinicians, patients and their caregivers for BP goals should be utilized when the patient has a limited life expectancy or is institutionalized due to high burden of frailty and comorbidity with limited life expectancy. However, no strict cut-off values or therapeutic approach was addressed, unlike the detailed management instructions provided by 2023 ESH that provides separate guidance for four different age ranges, ranging from 18–64 to over 80 years of age.

KDIGO reiterated in 2024 the suggestion to aim at a target SBP <120 mmHg for patients with CKD, when tolerated, using standardized office BP measurement [19].

All guidelines recommend initiation of single pill once-daily combination therapy to improve medication adherence, except for patients with frailty or symptomatic orthostatic hypotension (Class I recommendation, Level B of evidence). A third agent is added if BP optimization is not reached, followed by dose titration to maximally tolerated doses. Initially recommended anti-hypertensive medications include angiotensin-converting enzyme inhibitors (ACEI) or angiotensin-receptor antagonists (ARB), long-acting dihydropyridine type calcium channel blockers and thiazide-type diuretics. The 2025 AHA/ACC guidelines consider treatment with a single first-line once daily agent with close follow-up an option for adults with stage 1 hypertension (SBP 130–39 mmHg, DBP 80–89 mmHg). This is recommended to be ACEI or ARB for people with CKD or with type 2 diabetes, a change from the prior edition which stated that they be considered for CKD G3–G5 or A3 albuminuria.

The approach towards resistant hypertension is similar between guidelines. First, address drug adherence and eliminate potential causes of pseudo-resistant hypertension. Patients with truly resistant hypertension should receive MRAs if not contraindicated. If contraindicated or failed, potential additional pharmacotherapeutic options include beta-blockers, alpha-blockers, amiloride, central-acting sympatholytic agents, endothelin receptor antagonists or direct vasodilators.

The diagnosis, follow-up and treatment of elevated BP during pregnancy are challenging. Under physiological conditions BP measurements are expected to reach a nadir by 20th gestational week followed by a steady increase towards term [38]. The 2025 AHA/ACC mainly refers to a 2020 American College of Obstetricians and Gynecologists document [39]. The diagnosis and follow-up of gestational hypertension is mostly mediated via antenatal visits and in-office measurements such as HBPM or ABPM are not validated in pregnant individuals [40]. Classification of hypertension among pregnant women is based upon the presence of elevated BP prior to pregnancy, gestational week at diagnosis or persistence of hypertension at postpartum period. The guidelines are aligned regarding the potential harm of anti-hypertensive treatment options including ACEI, ARB, direct renin inhibitors, MRA and atenolol. Similarly, guidelines recommend preferential use of dihydropyridine type calcium channel blockers, especially extended-release nifedipine, labetalol and methyldopa, along with lifestyle interventions, with a target SBP <140 mmHg and DBP <90 mmHg (Class I recommendation, Level A of evidence). Moreover, guidelines emphasize the use of low-dose aspirin (81 mg) to prevent pre-eclampsia in high-risk patients with gestational hypertension (Class I recommendation, Level B of evidence). The 2024 ESC guidelines recommend preventive measures including low-to-moderate intensity physical exercise and 0.5–2 g/day oral calcium supplementation for high-risk patients.

## INTERVENTIONAL THERAPEUTIC ALTERNATIVES

Renal denervation aiming to disrupt afferent and efferent sympathetic nerves is a novel Food and Drug Administration–approved therapeutic approach towards the management of hypertension. Randomized clinical trials comparing the efficacy of such an interventional approach over sham operation among patients receiving anti-hypertensive medications or not have reported slight reductions in ambulatory SBP and DBP (up to 7.5/5 mmHg) [41–44]. Long term follow-up data for the efficacy of renal denervation on BP control was reported for over 3 years [45, 46] with a study reporting data up to 10 years in patients with resistant hypertension [47]. Of note, procedural complications including femoral access-related vascular complications or renal artery stenosis or dissection are rarely reported [48]. On the other hand, a meta-analysis study involving of 2478 hypertensive patients from 10 clinical trials reported only modest clinical benefit of renal denervation: a mean 4.4 mmHg decline in SBP and 2.6 mmHg decline in DBP in 24-h ABPM, with no significant difference in all-cause mortality, vascular complications, altered renal function or hypertensive crisis episodes [49]. Overall, all three recent guidelines acknowledge renal denervation as a valid but not initial therapeutic approach for the management of arterial hypertension among patients with resistant hypertension or patients experiencing intolerable medication-related adverse effects after careful discussion of the risks and benefits except for patients with low eGFR (<40 mL/min/1.73 m^2^) in whom it is contraindicated.

## AREAS FOR FUTURE CONSIDERATIONS

The 2025 AHA/ACC guidelines list some evidence gaps and future directions. These include the need for population management strategies to identify those with undiagnosed or uncontrolled hypertension to focus resources. Although not specifically mentioned, artificial intelligence may be useful for this purpose [50, 51]. They also point to the lack of evidence to support BP targets for diastolic hypertension; the need to define CVD endpoints for use outside academic centers, such as by pragmatic trial designs; the need for accurate BP measurement and to establish whether white-coat hypertension carries additional long-term risks; and what is the optimal combinations of medications dosed as separate agents to improve adherence and effectiveness. Finally, they point to the need to better understand and treat hypertension in pregnancy.

We may add additional items that should be addressed by future guidelines, such as seasonal variation in BP and digital interventions. There are significant inter-seasonal differences in BP reading, with an increase of 2–10 mmHg in SBP and 1–5 mmHg in DBP [52, 53]. Observational cohorts have shown higher rates of CVD and mortality with cold season–induced BP elevations [54–56] along with increased future cardiovascular events in patients exhibiting such high inter-seasonal BP variation [57]. Generalization of HBPM may optimize care across seasons.

Digital interventions may be used to promote lifestyle changes. A meta-analysis 12 clinical studies evaluating the effectiveness of phone-based applications targeting lifestyle interventions has illustrated beneficial effects as evidenced by larger declines in systolic [mean difference (MD) –2.91 mmHg, 95% confidence interval (CI) –4.11, –1.71] and diastolic (MD –1.13 mmHg, 95% CI –1.91, –0.35) compared with controls [58].

Although the screening and proper addressing of CKD have strongly been suggested in the 2025 AHA/ACC guidelines with strong emphasis, there is currently lack of strong clinical data on end-stage kidney disease patients undergoing kidney replacement therapies including hemodialysis and peritoneal dialysis. However, the major pathophysiological differences between essential hypertension and hypertension in end-stage kidney disease (ESKD) patients should be properly addressed as crucial mechanisms include dialysate composition and prescription, secondary hyperparathyroidism, use of erythropoiesis-stimulating agents and increased arterial stiffness [59]. The importance of volume control in BP management among such populations was evident from a meta-analysis study illustrating a mean 2.7 mmHg SBP reduction with lung ultrasound-guided volume management [60] with a more recent randomized controlled trial of 71 patients indicating significant benefit of such an approach (–6.6 ± 9.6 mmHg vs −0.7 ± 12.1 mmHg) compared with standard of care [61]. A recent prospective randomized clinical trial has illustrated that reduction of dialysate sodium concentration from 140 to 138 mmol/L leads to significant decline in pre-dialysis SBP and interdialytic weight gain [62]. Similarly, more intensified, even daily, hemodialysis sessions may induce better BP control [63, 64]. Moreover, the knowledge on pharmacotherapeutic perspective for ESKD is highly scarce with limited number of studies on beta-blockers including atenolol [65], calcium channel blockers including amlodipine [66, 67], MRAs including spironolactone [68], and RAAS inhibitors including fosinopril and candesartan [69, 70]. An additional concern while administering pharmacotherapies for hypertension among patients undergoing kidney replacement therapy is the potential dialyzability of medications, as certain beta-blockers including atenolol and metoprolol and RAAS inhibitors such as captopril, ramipril and lisinopril show rapid clearance with hemodialysis [71]. However, there is a strong need for future clinical trials in such high-risk population with limited available clinical data with inconsistent results.

## CLOSING REMARKS

The 2025 AHA/ACC hypertension guidelines follow quickly on from the 2023 ESH and 2024 ESC guidelines. It is reassuring to see that a strict evidence-based approach to evidence synthesis and guideline production is resulting in much closer harmonization of recommendations over time. In short, the similarities between the guidelines are becoming more evident than the differences. Overall, 2025 AHA/ACC supports pharmacotherapy earlier and achieving lower BP targets than other recent guidelines. There are also differences in the concept of hypertension which can be traced to the recommended or suggested attitude towards pharmacotherapy of SBP ≥130 mmHg or DBP ≥80 mmHg: 2025 AHA/ACC labels it hypertension and supports pharmacotherapy for all, independently of cardiovascular risk, while 2024 ESC and 2023 ESH use alternative terms (high normal or elevated BP) for BP values (i.e. ≥130–139 or ≥80–89 mmHg) that require pharmacotherapy only in high-risk groups. Differences are also found in guidance to lower SBP <120 mmHg: while the 2023 ESH guideline recommends avoiding actively aiming for SBP <120 mmHg or DBP <70 mmHg, 2025 AHA/ACC suggest a target SBP <120 mmHg, in line with 2024 KDIGO guidance for people with CKD. Although the authors of this manuscript believe that targeting SBP below 120 mmHg may be associated with better clinical outcomes as indicated by the 2024 KDIGO and 2025 AHA/ACC guidelines based upon the subgroup analysis data of the SPRINT (Systolic Blood Pressure Intervention Trial) trial indicating lower all-cause mortality (hazard ratio 0.72, 95% CI 0.53–0.99) [72], it would be desirable that guidelines further converge on such key issues as nomenclature and therapeutic targets.

For healthcare professionals treating patients with CKD, it is heartening to see that all three guidelines emphasize the importance of diagnosing CKD, not just by estimating GFR, but also by measuring albuminuria. Thus, all guidelines recommend screening for CKD in patients with high BP and hypertension. The high risk of cardiovascular events of patients with CKD is recognized in all three guidelines and is reflected in treatment decisions. With CKD projected to become the fifth leading cause of death by 2040, and third in many European countries, these are timely, important and very welcome developments [73, 74]. The onus is now on all of us who care for patients with CKD, to ensure diagnosis and correct treatment of hypertension by implementing any of these guidelines to improve their outcomes.

## ETHICS STATEMENT

This review is based entirely on previously published literature. No new data involving humans or animals were collected. All sources are properly cited, and the authors declare the work is original, ethical and free from conflicts of interest.

## Data Availability

No new data were generated in this manuscript.

## References

[1] Global Burden of Cardiovascular Diseases and Risks 2023 Collaborators . Global, regional, and national burden of cardiovascular diseases and risk factors in 204 countries and territories, 1990-2023. J Am Coll Cardiol. 2025;86:2167–243. 10.1016/j.jacc.2025.08.015 40990886

[2] GBD 2023 Chronic Kidney Disease Collaborators . Global, regional, and national burden of chronic kidney disease in adults, 1990-2023, and its attributable risk factors: a systematic analysis for the global burden of disease study 2023. Lancet. 2025;406:2461–82. 10.1016/S0140-6736(25)01853-7 41213283

[3] Jones DW, Ferdinand KC, Taler SJ et al. AHA/ACC/AANP/AAPA/ABC/ACCP/ACPM/AGS/AMA/ASPC/NMA/PCNA/SGIM guideline for the prevention, detection, evaluation and management of high blood pressure in adults: a report of the American College of Cardiology/American Heart Association joint committee on clinical practice guidelines. Circulation. 2025;86(18):1567–1678. 10.1016/j.jacc.2025.10.006 40815242

[4] Whelton PK, Carey RM, Aronow WS et al. 2017 ACC/AHA/AAPA/ABC/ACPM/AGS/APhA/ASH/ASPC/NMA/PCNA guideline for the prevention, detection, evaluation, and management of high blood pressure in adults: executive summary: a report of the American College of Cardiology/American Heart Association task force on clinical practice guidelines. Circulation. 2018;138:e426–83. 10.1161/CIR.0000000000000597 30354655

[5] McEvoy JW, McCarthy CP, Bruno RM et al. 2024 ESC guidelines for the management of elevated blood pressure and hypertension: developed by the task force on the management of elevated blood pressure and hypertension of the European Society of Cardiology (ESC) and endorsed by the European Society of Endocrinology (ESE) and the European Stroke Organisation (ESO). Eur Heart J. 2024;45:3912–4018.

[6] Mancia G, Kreutz R, Brunström M et al. 2023 ESH guidelines for the management of arterial hypertension the task force for the management of arterial hypertension of the European Society of Hypertension: endorsed by the International Society Of Hypertension (ISH) and the European Renal Association (ERA). J Hypertens. 2023;41:1874–2071. 10.1097/HJH.0000000000003480 37345492

[7] Dominiczak AF, Delles C. Hypertension in 2025: are we ready for bold precision public health approaches worldwide?. Nat Rev Cardiol. 2025;22:607–8. 10.1038/s41569-025-01192-5 40813924

[8] Joynt Maddox KE, Elkind MSV, Aparicio HJ et al. Forecasting the burden of cardiovascular disease and stroke in the United States through 2050—prevalence of risk factors and disease: a presidential advisory from the American Heart Association. Circulation. 2024;150:e65–88. 10.1161/CIR.0000000000001256 38832505

[9] GBD 2023 Disease and Injury and Risk Factor Collaborators . Burden of 375 diseases and injuries, risk-attributable burden of 88 risk factors, and healthy life expectancy in 204 countries and territories, including 660 subnational locations, 1990-2023: a systematic analysis for the global burden of disease study 2023. Lancet. 2025;406:1873–922. 10.1016/S0140-6736(25)01637-X PMC12535840 41092926

[10] Sarafidis PA, Li S, Chen SC et al. Hypertension awareness, treatment, and control in chronic kidney disease. Am J Med. 2008;121:332–40. 10.1016/j.amjmed.2007.11.025 18374693

[11] Global, regional, and national prevalence of kidney failure with replacement therapy and associated aetiologies, 1990-2023: a systematic analysis for the global burden of disease study 2023. Lancet Glob Health. 2025;13:e1378–95. 10.1016/S2214-109X(25)00198-6 40712611

[12] Foundation ACoC . Methodology Manual and Policies from the ACCF/AHA Task Force on Practice Guidelines. American College of Cardiology Foundation and American Heart Association. 2010. http://my.americanheart.org/idc/groups/ahamah-public/@wcm/@sop/documents/downloadable/ucm_319826.pdf, date last accessed, 5 December 2026.

[13] World Health Organization . Global Report on Hypertension: The Race Against a Silent Killer. Geneva, Switzerland: World Health Organization, 2023, 1–276.

[14] Morton S, Berg A, Levit L et al. Institute of Medicine (US) Committee on Standards for Systematic Reviews of Comparative Effectiveness Research. Eden J, Levit L, Morton S, (eds). Finding What Works in Health Care: Standards for Systematic Reviews. Washington (DC): National Academies Press (US), 2011. 24983062

[15] Shimbo D, Abdalla M, Falzon L et al. Studies comparing ambulatory blood pressure and home blood pressure on cardiovascular disease and mortality outcomes: a systematic review. J Am Soc Hypertens. 2016;10:224–34. 10.1016/j.jash.2015.12.013 PMC4788568 26822864

[16] Panagiotakos D, Antza C, Kotsis V. Ambulatory and home blood pressure monitoring for cardiovascular disease risk evaluation: a systematic review and meta-analysis of prospective cohort studies. J Hypertens. 2024;42:1–9. 10.1097/HJH.0000000000003557 37702566

[17] Agarwal R, Bills JE, Hecht TJ et al. Role of home blood pressure monitoring in overcoming therapeutic inertia and improving hypertension control: a systematic review and meta-analysis. Hypertension. 2011;57:29–38. 10.1161/HYPERTENSIONAHA.110.160911 21115879

[18] Tucker KL, Sheppard JP, Stevens R et al. Self-monitoring of blood pressure in hypertension: a systematic review and individual patient data meta-analysis. PLoS Med. 2017;14:e1002389. 10.1371/journal.pmed.1002389 PMC5604965 28926573

[19] KDIGO 2024 clinical practice guideline for the evaluation and management of chronic kidney disease. Kidney Int. 2024;105:S117–314. 10.1016/j.kint.2023.10.018 38490803

[20] Azizi M, Sanghvi K, Saxena M et al. Ultrasound renal denervation for hypertension resistant to a triple medication pill (RADIANCE-HTN TRIO): a randomised, multicentre, single-blind, sham-controlled trial. Lancet. 2021;397:2476–86. 10.1016/S0140-6736(21)00788-1 34010611

[21] Carey RM, Calhoun DA, Bakris GL et al. Resistant hypertension: detection, evaluation, and management: a scientific statement from the American Heart Association. Hypertension. 2018;72:e53–90. 10.1161/HYP.0000000000000084 PMC6530990 30354828

[22] Shimbo D, Artinian NT, Basile JN et al. Self-measured blood pressure monitoring at home: a joint policy statement from the American Heart Association and American Medical Association. Circulation. 2020;142:e42–63. 10.1161/CIR.0000000000000803 32567342

[23] McManus RJ, Little P, Stuart B et al. Home and online management and evaluation of blood pressure (HOME BP) using a digital intervention in poorly controlled hypertension: randomised controlled trial. BMJ. 2021;372:m4858. 10.1136/bmj.m4858 PMC7814507 33468518

[24] Dimitriadis K, Pyrpyris N, Vakka A et al. Microalbuminuria as a prognostic marker in essential hypertension. Curr Med Chem. 2025;32:8855–84. 10.2174/0109298673348953250310044927 40143689

[25] Tandan N, Lavie CJ, Stewart MH et al. Secondary hypertension: evaluation and management. Curr Opin Cardiol. 2023;38:318–25. 10.1097/HCO.0000000000001059 37115960

[26] McDonagh TA, Metra M, Adamo M et al. 2023 focused update of the 2021 ESC guidelines for the diagnosis and treatment of acute and chronic heart failure. Eur Heart J. 2023;44:3627–39. 10.1093/eurheartj/ehad195 37622666

[27] Ortiz A, Arreola Guerra JM, Chan JCN et al. Preventing chronic kidney disease and maintaining kidney health: conclusions from a kidney disease: improving global outcomes (KDIGO) controversies conference. Kidney Int. 2025;108:555–71. 10.1016/j.kint.2025.04.005 PMC12646115 40536455

[28] Ortiz A, Yanagita M, Yokoi H et al. Evolving strategies for early diagnosis, proactive prevention and treatment of CKD. Nephrol Dial Transplant. 2025;41(3):418–427. 10.1093/ndt/gfaf151 40795360

[29] Vinceti M, Filippini T, Crippa A et al. Meta-analysis of potassium intake and the risk of stroke. J Am Heart Assoc. 2016;5(10):e004210. 10.1161/JAHA.116.004210 PMC5121516 27792643

[30] Zhang Z, Cogswell ME, Gillespie C et al. Association between usual sodium and potassium intake and blood pressure and hypertension among U.S. adults: NHANES 2005-2010. PLoS One. 2013;8:e75289. 10.1371/journal.pone.0075289 PMC3794974 24130700

[31] Gan L, Zhao B, Inoue-Choi M et al. Sex-specific associations between sodium and potassium intake and overall and cause-specific mortality: a large prospective U.S. cohort study, systematic review, and updated meta-analysis of cohort studies. BMC Med. 2024;22:132. 10.1186/s12916-024-03350-x PMC10960470 38519925

[32] SCORE2-OP risk prediction algorithms: estimating incident cardiovascular event risk in older persons in four geographical risk regions. Eur Heart J. 2021;42:2455–67. 10.1093/eurheartj/ehab312 PMC8248997 34120185

[33] SCORE2 risk prediction algorithms: new models to estimate 10-year risk of cardiovascular disease in Europe. Eur Heart J. 2021;42:2439–54. 10.1093/eurheartj/ehab309 PMC8248998 34120177

[34] Scheuermann B, Brown A, Colburn T et al. External validation of the American Heart Association PREVENT cardiovascular disease risk equations. JAMA Netw Open. 2024;7:e2438311. 10.1001/jamanetworkopen.2024.38311 PMC11470385 39392632

[35] Khan SS, Matsushita K, Sang Y et al. Development and validation of the American Heart Association’s PREVENT equations. Circulation. 2024;149:430–49. 10.1161/CIRCULATIONAHA.123.067626 PMC10910659 37947085

[36] D’Agostino RB Sr, Vasan RS, Pencina MJ et al. General cardiovascular risk profile for use in primary care: the Framingham heart study. Circulation. 2008;117:743–53. 10.1161/CIRCULATIONAHA.107.699579 18212285

[37] Wright JT Jr, Williamson JD, Whelton PK et al. A randomized trial of intensive versus standard blood-pressure control. N Engl J Med. 2015;373:2103–16. 10.1056/NEJMoa1511939 PMC4689591 26551272

[38] Ishikuro M, Obara T, Metoki H et al. Blood pressure measured in the clinic and at home during pregnancy among nulliparous and multiparous women: the BOSHI study. Am J Hypertens. 2013;26:141–8. 10.1093/ajh/hps002 23382338

[39] Gestational hypertension and preeclampsia: ACOG practice bulletin, number 222. Obstet Gynecol. 2020;135:e237–60. 10.1097/AOG.0000000000003891 32443079

[40] Chappell LC, Tucker KL, Galal U et al. Effect of self-monitoring of blood pressure on blood pressure control in pregnant individuals with chronic or gestational hypertension: the BUMP 2 randomized clinical trial. JAMA. 2022;327:1666–78. 10.1001/jama.2022.4726 PMC9066282 35503345

[41] Böhm M, Kario K, Kandzari DE et al. Efficacy of catheter-based renal denervation in the absence of antihypertensive medications (SPYRAL HTN-OFF MED Pivotal): a multicentre, randomised, sham-controlled trial. Lancet. 2020;395:1444–51. 10.1016/S0140-6736(20)30554-7 32234534

[42] Weber MA, Kirtane AJ, Weir MR et al. The REDUCE HTN: REINFORCE: randomized, sham-controlled trial of bipolar radiofrequency renal denervation for the treatment of hypertension. JACC Cardiovasc Interv. 2020;13:461–70. 10.1016/j.jcin.2019.10.061 32081240

[43] Kandzari DE, Böhm M, Mahfoud F et al. Effect of renal denervation on blood pressure in the presence of antihypertensive drugs: 6-month efficacy and safety results from the SPYRAL HTN-ON MED proof-of-concept randomised trial. Lancet. 2018;391:2346–55. 10.1016/S0140-6736(18)30951-6 29803589

[44] Azizi M, Schmieder RE, Mahfoud F et al. Endovascular ultrasound renal denervation to treat hypertension (RADIANCE-HTN SOLO): a multicentre, international, single-blind, randomised, sham-controlled trial. Lancet. 2018;391:2335–45. 10.1016/S0140-6736(18)31082-1 29803590

[45] Rader F, Kirtane AJ, Wang Y et al. Durability of blood pressure reduction after ultrasound renal denervation: three-year follow-up of the treatment arm of the randomised RADIANCE-HTN SOLO trial. EuroIntervention. 2022;18:e677–85. 10.4244/EIJ-D-22-00305 PMC10241283 35913759

[46] Mahfoud F, Kandzari DE, Kario K et al. Long-term efficacy and safety of renal denervation in the presence of antihypertensive drugs (SPYRAL HTN-ON MED): a randomised, sham-controlled trial. Lancet. 2022;399:1401–10. 10.1016/S0140-6736(22)00455-X 35390320

[47] Al Ghorani H, Kulenthiran S, Recktenwald MJM et al. 10-year outcomes of catheter-based renal denervation in patients with resistant hypertension. J Am Coll Cardiol. 2023;81:517–9. 10.1016/j.jacc.2022.11.038 36725181

[48] Townsend RR, Walton A, Hettrick DA et al. Review and meta-analysis of renal artery damage following percutaneous renal denervation with radiofrequency renal artery ablation. EuroIntervention. 2020;16:89–96. 10.4244/EIJ-D-19-00902 32038027

[49] Vukadinović D, Lauder L, Kandzari DE et al. Effects of catheter-based renal denervation in hypertension: a systematic review and meta-analysis. Circulation. 2024;150:1599–611. 10.1161/CIRCULATIONAHA.124.069709 PMC11560572 39355923

[50] Chaikijurajai T, Laffin LJ, Tang WHW. Artificial intelligence and hypertension: recent advances and future outlook. Am J Hypertens. 2020;33:967–74. 10.1093/ajh/hpaa102 PMC7608522 32615586

[51] Cho JS, Park J-H. Application of artificial intelligence in hypertension. Clin Hypertens. 2024;30:11. 10.1186/s40885-024-00266-9 PMC11061896 38689376

[52] Narita K, Hoshide S, Kario K. Seasonal variation in blood pressure: current evidence and recommendations for hypertension management. Hypertens Res. 2021;44:1363–72. 10.1038/s41440-021-00732-z 34489592

[53] Barbosa ECD, Farina GS, Basso CS et al. Seasonal variation in blood pressure: what is still missing?. Front Cardiovasc Med. 2023;10:1233325. 10.3389/fcvm.2023.1233325 PMC10469506 37663410

[54] Yang L, Li L, Lewington S et al. Outdoor temperature, blood pressure, and cardiovascular disease mortality among 23 000 individuals with diagnosed cardiovascular diseases from China. Eur Heart J. 2015;36:1178–85. 10.1093/eurheartj/ehv023 PMC4430682 25690792

[55] Gasparrini A, Guo Y, Hashizume M et al. Mortality risk attributable to high and low ambient temperature: a multicountry observational study. Lancet. 2015;386:369–75. 10.1016/S0140-6736(14)62114-0 PMC4521077 26003380

[56] Aubinière-Robb L, Jeemon P, Hastie CE et al. Blood pressure response to patterns of weather fluctuations and effect on mortality. Hypertension. 2013;62:190–6. 10.1161/HYPERTENSIONAHA.111.00686 23648702

[57] Hanazawa T, Asayama K, Watabe D et al. Association between amplitude of seasonal variation in self-measured home blood pressure and cardiovascular outcomes: HOMED-BP (hypertension objective treatment based on measurement by electrical devices of blood pressure) study. J Am Heart Assoc. 2018;7(10):e008509. 10.1161/JAHA.117.008509 PMC6015300 29728372

[58] Lindsay-Perez A, Jurdon R, King T et al. A systematic review and meta-analysis of digital interventions targeting lifestyle factors in patients with hypertension. J Hum Hypertens. 2025;39(10):690–700. 10.1038/s41371-025-01051-3 PMC12500467 40764400

[59] Santos SF, Peixoto AJ. Hypertension in dialysis. Curr Opin Nephrol Hypertens. 2005;14:111–8. 10.1097/00041552-200503000-00005 15687836

[60] Covic A, Ciumanghel AI, Siriopol D et al. Value of bioimpedance analysis estimated “dry weight” in maintenance dialysis patients: a systematic review and meta-analysis. Int Urol Nephrol. 2017;49:2231–45. 10.1007/s11255-017-1698-4 28965299

[61] Loutradis C, Sarafidis PA, Ekart R et al. The effect of dry-weight reduction guided by lung ultrasound on ambulatory blood pressure in hemodialysis patients: a randomized controlled trial. Kidney Int. 2019;95:1505–13. 10.1016/j.kint.2019.02.018 31027889

[62] Varda L, Piko N, Bevc S et al. Effect of Different dialysate sodium concentrations on blood pressure in chronic haemodialysis patients: a randomized study. Blood Purif. 2025;54:576–89. 10.1159/000546469 40609518

[63] Woods JD, Port FK, Orzol S et al. Clinical and biochemical correlates of starting “daily” hemodialysis. Kidney Int. 1999;55:2467–76. 10.1046/j.1523-1755.1999.00493.x 10354296

[64] Pierratos A, Ouwendyk M, Francoeur R et al. Nocturnal hemodialysis: three-year experience. J Am Soc Nephrol. 1998;9:859–68. 10.1681/ASN.V95859 9596084

[65] Agarwal R, Sinha AD, Pappas MK et al. Hypertension in hemodialysis patients treated with atenolol or lisinopril: a randomized controlled trial. Nephrol Dial Transplant. 2014;29:672–81. 10.1093/ndt/gft515 PMC3938300 24398888

[66] Tepel M, Hopfenmueller W, Scholze A et al. Effect of amlodipine on cardiovascular events in hypertensive haemodialysis patients. Nephrol Dial Transplant. 2008;23:3605–12. 10.1093/ndt/gfn304 PMC2568006 18511605

[67] Mugendi GA, Mutua FM, Natale P et al. Calcium channel blockers for people with chronic kidney disease requiring dialysis. Cochrane Database Syst Rev. 2020;10:Cd011064. 10.1002/14651858.CD011064.pub2 PMC8094736 33000470

[68] Charytan DM, Himmelfarb J, Ikizler TA et al. Safety and cardiovascular efficacy of spironolactone in dialysis-dependent ESRD (SPin-D): a randomized, placebo-controlled, multiple dosage trial. Kidney Int. 2019;95:973–82. 10.1016/j.kint.2018.08.034 PMC6431563 30473139

[69] Zannad F, Kessler M, Lehert P et al. Prevention of cardiovascular events in end-stage renal disease: results of a randomized trial of fosinopril and implications for future studies. Kidney Int. 2006;70:1318–24. 10.1038/sj.ki.5001657 16871247

[70] Takahashi A, Takase H, Toriyama T et al. Candesartan, an angiotensin II type-1 receptor blocker, reduces cardiovascular events in patients on chronic haemodialysis—a randomized study. Nephrol Dial Transplant. 2006;21:2507–12. 10.1093/ndt/gfl293 16766543

[71] Bansal N, Artinian NT, Bakris G et al. Hypertension in patients treated with in-center maintenance hemodialysis: current evidence and future opportunities: a scientific statement from the American Heart Association. Hypertension. 2023;80:e112–22. 10.1161/HYP.0000000000000230 37092336

[72] Cheung AK, Rahman M, Reboussin DM et al. Effects of intensive BP control in CKD. J Am Soc Nephrol. 2017;28:2812–23. 10.1681/ASN.2017020148 PMC5576945 28642330

[73] Ferro CJ, Wanner C, Luyckx V et al. A call for urgent action on chronic kidney disease across Europe. Lancet Reg Health Eur. 2025;54:101347. 10.1016/j.lanepe.2025.101347 PMC12182384 40548327

[74] Ferro CJ, Wanner C, Luyckx V et al. ABCDE to identify and prevent chronic kidney disease: a call to action. Nephrol Dial Transplant. 2025;40:1786–98. 10.1093/ndt/gfaf057 PMC12451690 40107862

[75] Jones DW, Ferdinand KC, Taler SJ et al. 2025 AHA/ACC/AANP/AAPA/ABC/ACCP/ACPM/AGS/AMA/ASPC/NMA/PCNA/SGIM guideline for the prevention, detection, evaluation and management of high blood pressure in adults: a report of the American College of Cardiology/American Heart Association joint committee on clinical practice guidelines. Circulation. 2025;152:e114–218. 10.1161/CIR.0000000000001356 40811497

